# Different Nuclear Architecture in Human Sperm According to Their Morphology

**DOI:** 10.3390/genes15040464

**Published:** 2024-04-07

**Authors:** Nino-Guy Cassuto, Nesrine Ogal, Said Assou, Lea Ruoso, Eli-Jonathan Rogers, Miguel-José Monteiro, Daniel Thomas, Jean-Pierre Siffroi, Alexandre Rouen

**Affiliations:** 1 ART Unit, Drouot Laboratory, 75009 Paris, France; learuoso@labodrouot.com (L.R.); miguel@nanovare.com (M.-J.M.); daniel@mojofertility.com (D.T.); 2 Department of Medical Genetics, Armand-Trousseau Hospital, AP-HP, INSERM Unit U933, Sorbonne University, 75012 Paris, France; ogal.nesrine@hotmail.com (N.O.); eroge10@u.rochester.edu (E.-J.R.); jean-pierre.siffroi@aphp.fr (J.-P.S.); 3 Institute for Regenerative Medicine and Biotherapy, University Montpellier, INSERM, CHU Montpellier, 34295 Montpellier, France; said.assou@inserm.fr; 4 Maternity of Bluets, Medically Assisted Reproduction Service, 75012 Paris, France; 5 AP-HP, Hôtel-Dieu, Sleep and Vigilance Center, Université Paris Cité, VIFASOM, ERC 7330, 75010 Paris, France

**Keywords:** nuclear architecture, telomere, spermatozoa morphology, high magnification, fluorescent in situ hybridization

## Abstract

Human sperm parameters serve as a first step in diagnosing male infertility, but not in determining the potential for successful pregnancy during assisted reproductive technologies (ARTs) procedures. Here, we investigated the relationship between sperm head morphology at high magnification, based on strict morphologic criteria, and the nuclear architecture analyzed by fluorescence in situ hybridization (FISH). We included five men. Two of them had an elevated high-magnification morphology score of 6 points (Score 6) indicating high fertility potential, whereas three had a low score of 0 points (Score 0), indicating low fertility potential. We used FISH to study the inter-telomeric distance and the chromosomal territory area of chromosome 1 (Chr. 1). We then compared these two parameters between subjects with high and low scores. FISH data analysis showed that the inter-telomeric distance (ITD) and chromosomal territory area (CTA) of Chr. 1 were significantly higher in subjects with low scores (score 0) than high scores (score 6). Our results suggest that (i) there is a link between nuclear architecture and sperm head abnormalities, particularly vacuoles; and (ii) it is possible to select spermatozoa with normal nuclear architecture, which might indirectly explain the positive ART outcomes observed with this technique.

## 1. Introduction

In vitro fertilization was developed in 1978 to address tubal factor infertility [1], while intracytoplasmic sperm injection (ICSI) was introduced in 1992 to overcome male factor infertility [2]. Thereafter, evidence began to emerge that the selection of spermatozoon before injection into the oocyte has a significant impact on ICSI outcomes [3]. Choosing spermatozoa remains a pivotal stage, influencing the cascade of fertilization processes and early embryo development. The main challenge arises from the varied characteristics of spermatozoa within one ejaculation. While microscopy allows for selection based on the appearance and motility of spermatozoa, it is worth noting that morphology primarily represents a superficial characteristic.

In 2009, Cassuto et al. proposed a sperm classification score based on high magnification analysis [4]. This score takes into account the shape and size of the head, the shape of the base, and the presence or absence of head vacuoles. It ranges from a score of 6 (high quality) to a score of 0 (low quality). Briefly, spermatozoa with a total score of 6 points display a normal head shape (normal head, with symmetrical nucleus, no extrusion and/or no invagination of the nuclear membrane = 2 points) without any vacuole (3 points), and normal base (the third inferior part of the sperm head to the neck, where the centrosome is localized = 1 point). The spermatozoa with a total score of 0 (0 points) display head shape abnormalities, vacuoles, and an abnormal base. Fertilization rates and the number of good-quality blastocysts at day 5 (according to Gardner’s classification) are significantly higher when using score 6 spermatozoa [4,5]. Since then, several studies have confirmed that high-magnification morphological spermatozoa correlate with higher blastulation rate [6,7,8].

**Figure 1:**
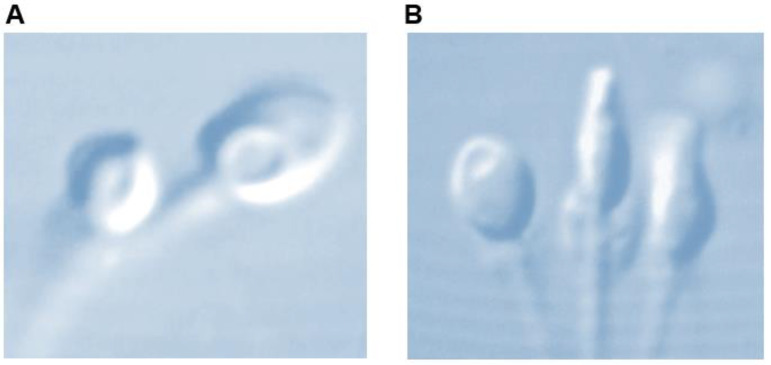
Sperm head vacuole at high magnification (×6100). (**A**) Post acrosomal, (**B**) acrosomal.

Utilizing Nomarski and differential interference contrast (DIC) polarization techniques on an optical inverted microscope equipment with a glass-bottomed Petri dish enables the observation of live spermatozoa at a high magnification level (×6100). This enables the thorough examination of the strict morphology of the sperm head and the identification of any possible vacuoles (Figure 1). Many investigations mention the presence of these vacuoles on the sperm head [9,10,11,12].

Recently, a link was established between morphology and DNA quality, as spermatozoa possess identical DNA sequences yet exhibit diverse epigenetic profiles. For this purpose, we profiled the global DNA methylation in sperm and reported a strong correlation between sperm morphology and the expression and methylation status of ten genes [13]. These genes constitute a distinctive sperm signature, offering a novel approach for analyzing sperm during assisted reproductive technologies (ARTs) and investigating male infertility.

**Figure 2:**
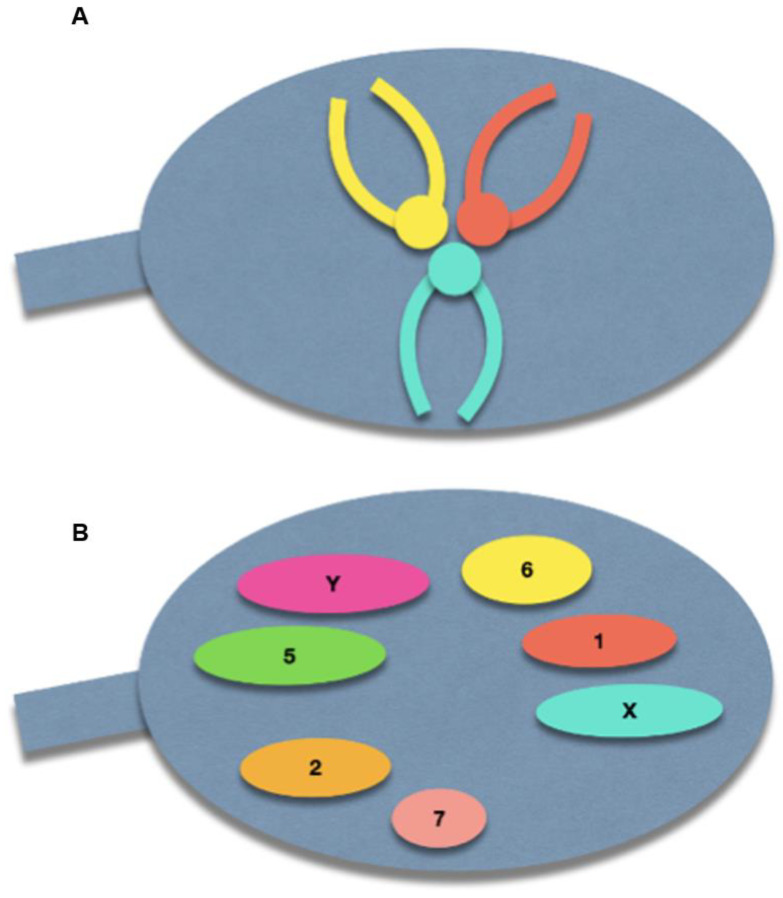
Schematic illustration of the normal sperm architecture. (**A**) Centrally-located centromeres, with the telomeres bound to each other at the periphery of the nuclear membrane. (**B**) Preferential locations of each chromosome within the sperm nucleus.

The spermatozoon nucleus is characterized by a high degree of condensation compared with other cell types. Indeed, a spermatozoon contains only half of the genetic information compared to diploid cells, but has a nuclear volume of approximately 16 µm^3^, compared with 900 µm^3^ of lymphocyte nucleus [14]. The chromosomes within the spermatozoon are highly condensed, which has a suggested role in protecting paternal DNA before fertilization [15]. This tightly packed condensation is made possible by a specific chromosomal three-dimensional conformation: the centromeres are roughly located in the center of the nucleus, forming a structure called the chromocenter, and the telomeres of a given chromosome are covalently bound to each other near the periphery. Furthermore, chromosomes occupy distinct locations in the nucleus, called chromosome territories (Figure 2) [16,17,18]. Solov’eva et al. hypothesized that this covalent binding was responsible for the hairpin conformation of spermatic chromosomes, although the precise molecule remained to be established [19]. Zalensky et al. showed that this bound faded with increasing concentrations of heparin in the spermatozoa of different species, suggesting a protein-mediated interaction between telomeres, and between telomeres and the nuclear membrane [20].

It has been shown that spermatozoa with abnormal chromosomal content have an enlarged nucleus under confocal microscopy [21] and abnormal nuclear architecture [22]. This spermatic nuclear architecture can be evaluated by FISH and assessed by the two following parameters. (i) Inter-telomeric distance (ITD): the distance between telomeres (short arm, long arm) of a chromosome (chromosome 1, chosen for its length), normalized to the nucleus length from the base to the tip extremity; (ii) chromosomal territory area (CTA): the area of whole-chromosome painting fluorescence for chromosome 1, normalized to the total nuclear area.

Prompted by the aforementioned information, in the present study, we aimed to investigate the relationship between high-magnification sperm head morphology and its nuclear architecture.

## 2. Materials and Methods

The study was carried out in the ART Unit of the Drouot Laboratory, Paris, France, and Hospital Armand-Trousseau, AP-HP, Paris, France. The protocol was approved by the local ethics committee, the members of which are part of the Institutional Review Board (IRB) of the Société d’andrologie de langue Française (IORG0010678). All participants signed an informed consent form before inclusion in the study. They were informed that after finishing with clinical tests, their semen samples would be analyzed at high magnification. The patient’s confidentiality was ensured by data anonymization before analysis. This analysis did not lead to any additional costs for the patients and did not affect their treatment in any way. For the present study, 5 infertile men were enrolled: Subjects 1 and 2 had a high score of 6, suggesting high fertility potential, and subjects 3, 4, and 5 had a low score of 0. Semen samples were obtained by masturbation. Samples were incubated at 37 °C and then washed in Phosphate Buffered Saline (Eurobio, Paris, France) before being evaluated on a high-magnification microscope/micromanipulator (Olympus IX71, Olympus, Paris, France) and an Imagine Source camera (DMK33UP1300, Olympus, Paris, France). The spermatozoa were subsequently fixed in methanol and acetic acid (3:1) for 30 min at room temperature. They were then spread on microscope slides for further FISH analysis. Two types of probes were used: contiguous telomeric probes to calculate the ITD, and whole-chromosome painting probes to evaluate the CTA. Chromosome 1 (Chr. 1) was chosen for this study, based on our previous work [22].

For each spermatozoon, the ITD corresponded to the distance between the two telomere signals (short arm and long arm) of chromosome 1 over the nucleus length (measured from the intermediate piece insertion point to the tip) [22]. The CTA was calculated as the area of the whole chromosome 1 painting probe signal, over the whole nucleus area. Both were measured and calculated in 50 sperm cells.

The FISH assay was conducted as previously described by our team [23]. Briefly, fixed spermatozoa were decondensed by incubation for 2 m 30 at NaOH (1 M) before a hybridization step at 73 °C with the probes. FISH data were acquired with a fluorescence microscope (Olympus BX61), with a COHU 4912-5000 CCIR camera, and a ×100 oil immersion objective. With this configuration, 12 pixels in the image correspond to 1 µm. The open-source software Fiji was used for image analysis [24]. For each spermatozoon, the ITD corresponded to the distance between the two telomere signals (short arm and long arm) of chromosome 1 over the nucleus length (measured from the intermediate piece insertion point to the tip). The CTA was calculated as the area of the whole chromosome 1 painting probe signal, over the whole nucleus area. Both were measured and calculated in 50 sperm cells. These two parameters were then compared in subjects with high score (subjects 1 and 2) and low score (subjects 3, 4, and 5) by using the Mann–Whitney test, due to the non-normal distributions of data. A *p*-value < 0.05 was considered statistically significant. Data were then modeled using a decision tree algorithm to identify the features used by a machine learning model to distinguish between low- and high-score spermatozoa [25]. The splitting criterion for this decision tree was the Gini impurity, which indicates the likelihood of misclassification of new random data. It is calculated by subtracting the sum of the squared probabilities of each class (low or high) from 1, for each node, and by selecting the lowest value to minimize the Gini impurity of the split.

## 3. Results

**Figure 3:**
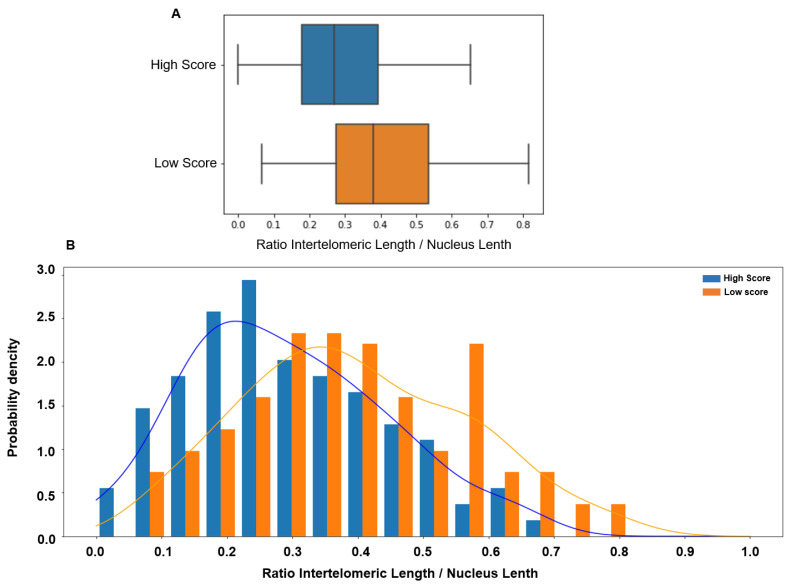
(**A**) The inter-telomeric distance (ITD; normalized to the whole nucleus length) is significantly higher in patients with low score in orange (median 0.379, 95% CI [0.06 0.73]) than in patients with high score in blue (median 0.269, 95% CI [0.00 0.59], *p* < 0.00001). (**B**) The ITD value distribution in patients with low (orange) and high (blue) scores shows a significant overlap.

Comparison of the ITD values between sperm samples with low and high score showed that the ITD was significantly higher in the low-score group (0.379 vs. 0.269, *p* < 0.00001). ITD value distribution in the low- and high-score groups showed a significant overlap (Figure 3).

**Figure 4:**
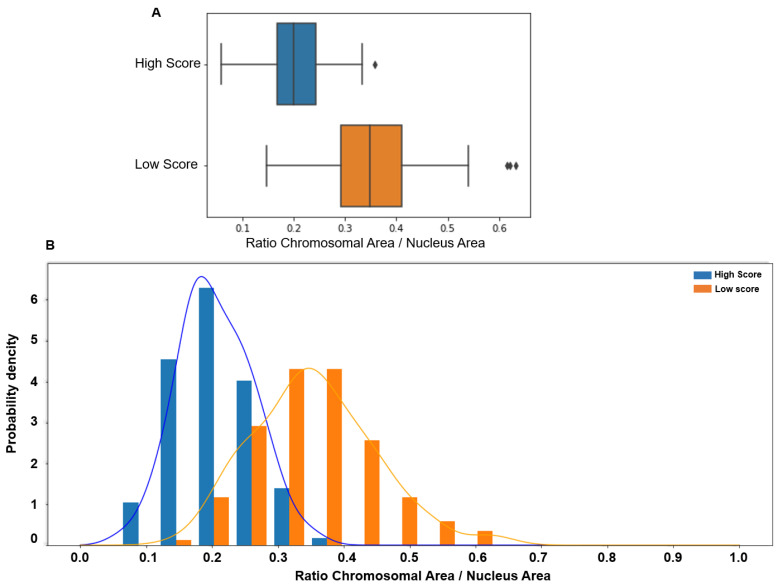
(**A**) The chromosomal territory area (CTA; normalized to the whole nucleus area) is significantly higher in patients with low (median: 0.349, 95% CI [0.17 0.54]) than high score (median 0.20, 95% CI [0.09 0.32], *p* < 0.00001). (**B**) The CTA value distribution in subjects with low (orange) and high (blue) scores shows a more limited overlap, with values > 0.4 observed only in spermatozoa with low scores.

Similarly, the CTA values were significantly higher in the lower than in the higher score group (0.349 vs. 0.2, *p* < 0.00001), with a tiny overlap compared with the ITD value. Indeed, CTA values > 0.4 were observed only in the low-score group (Figure 4).

**Figure 5:**
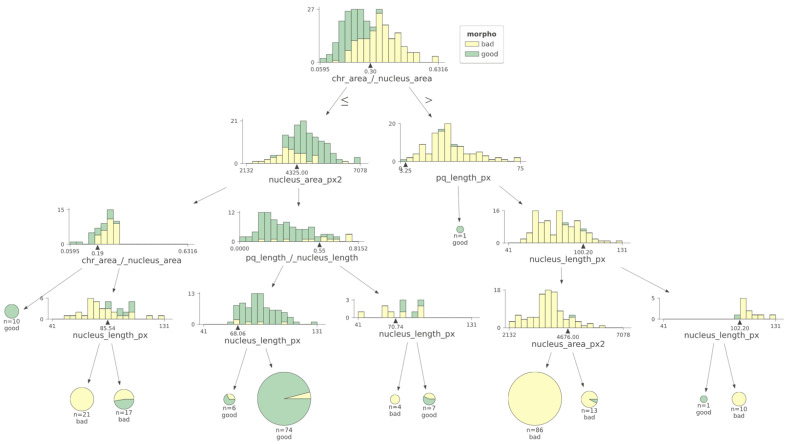
Using a machine learning decision tree algorithm, the most relevant criterion to differentiate low- versus high-score spermatozoa was CTA. Morpho: morphology. The black triangles represent the cut-off values determined for each parameter.

Analysis of the machine learning algorithm data indicates that CTA was the most relevant feature (i.e., the one that better discriminates between low- and high-score spermatozoa). The model also suggests that a CTA value of 0.30 was the best discriminant value. This value was within the interval of the median values of each class (0.20 and 0.349) and very close to their mean point (Figure 5).

## 4. Discussion

We previously showed that it is possible to assess sperm nuclear architecture using routine cytogenetic laboratory microscopes and probes. Normal sperm nuclear architecture is characterized by (1) significant nuclear condensation, (2) preferential locations for each chromosome, (3) positioning of the chromosome’s centromeres in the center of the nucleus forming a structure called the chromocenter, and (4) the telomeric extremities of a given chromosome bound to each other near the periphery of the nucleus [16,17,19]. This allows for a high degree of nuclear condensation in spermatozoa. Moreover, we reported that an altered nuclear architecture could be suspected based on the evaluation of two parameters: ITD and CTA. The distinct chromosomal architecture characterized by ITD and CTA may be associated with DNA fragmentation and potentially initiates the apoptosis process in spermatozoa scoring 0. Consequently, this could elucidate the decreased rates of fertilization and blastulation. Hence, preventing the selection of spermatozoa scoring 0 for injection in ICSI may be advisable to mitigate these outcomes. ITD could, therefore, be seen as a marker of physiological nuclear architecture and nuclear condensation, and as a layer epigenetic information. It has been hypothesized that the telomeres, joined as dimers in mammalian spermatozoa, may be involved in chromosome withdrawal during fertilization [17,26]. This would be made possible by a telomere–microtubule interaction, whose precise mechanism remains to be elucidated. An argument for this hypothesis is the impaired fertilization and early cleavage in telomerase -/- knockout mice [27]. On the other hand, the average length of sperm telomeres (STL) was notably shorter in infertile males compared to their fertile counterparts. Furthermore, a significant correlation was found between telomere length, sperm concentration, and DNA fragmentation, suggesting a potential link to fertility issues [28]. The sperm head within a single ejaculate displays variations in both ITD and CTA. This irregular nuclear chromosomal architecture, observed between a score of 0 and a score of 6, might be linked to additional defects such as reduced gene expression and altered epigenetic profiles, which collectively contribute to negative outcomes. Those spermatozoa lack the ability to reach a blastocyst by day 5 or to initiate a pregnancy. We see well through all those studies that the telomeres are sensible elements of the spermatozoa.

Chromosome 1 was chosen because of its length in the present study. Other chromosomes have been analyzed to study the spermatic nucleus [20,29,30,31,32,33]. Similar findings would be expected with other chromosomes, possibly in a less visible way, because of their inferior size. Employing the FISH method to examine telomere distribution, it was discovered that sperm nuclei from the infertile cohort displayed a greater surface area [34]. Based on Turner’s discovery, it is plausible to suggest that the distribution of telomeres in sperm nuclei from infertile individuals exhibits a larger surface area. This observation may be supported by the presence of nucleus invagination leading to vacuoles, which have detrimental effects [35].

Other teams have envisioned evaluating nuclear architecture in the fertility clinic [32]. Indeed, the methodology presented here is relatively simple and inexpensive and could be performed by any cytogenetics laboratory that routinely conducts fluorescent in situ hybridization assays. Further studies should aim at evaluating the clinical interest of evaluating spermatic nuclear architecture in infertile patients.

Using this methodology, we could detect an altered nuclear architecture in sperm samples from balanced chromosomal translocation carriers, and among specific sperm morphological classes after hypo-osmotic incubation [22,36]. Consequently, we proposed that a typical chromosomal arrangement, resembling its natural state, correlates with sperm fertility potential, while any deviations from this norm may induce an apoptosis mechanism, as evidenced in carriers of chromosomal translocations [37].

**Figure 6:**
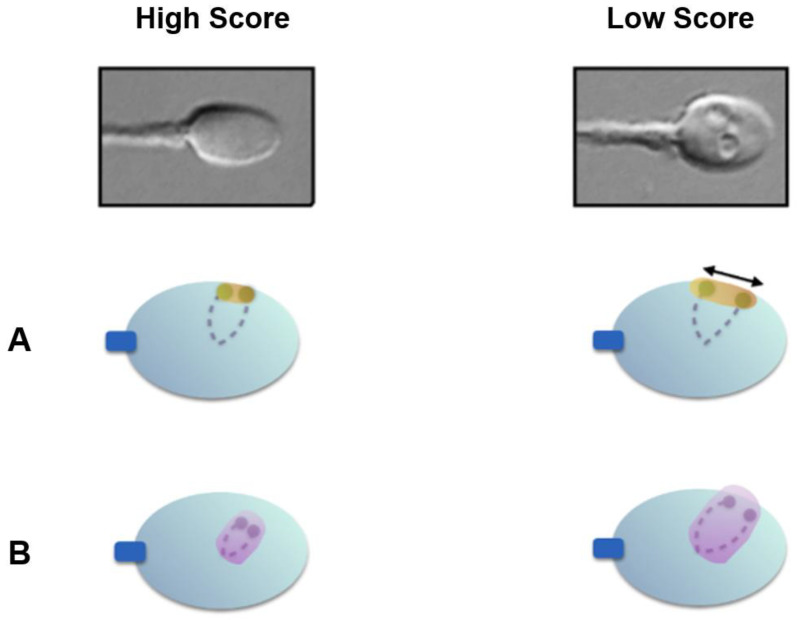
Nuclear architecture differences between high- and low-score spermatozoa. (**A**) Low-score spermatozoa exhibit significantly higher inter-telomeric distances; (**B**) low-score spermatozoa exhibit significantly higher chromosomal territory areas for chromosome 1.

In the present study, we used the same methodology to evaluate the nuclear architecture in subjects with low- and high-score spermatozoa. We further substantiated and validated the role of high magnification sperm morphology by demonstrating that ITD and CTA were higher in spermatozoa with low scores, suggesting their altered nuclear architecture (Figure 6), as compared to those with high scores.

One limitation of this study is the small number of subjects. However, the number of sperm cells analyzed in each subject was relatively high and the results observed were statistically significant. Nevertheless, these findings need to be confirmed with a larger sample size. Another limitation is the two-dimensional character of the analysis. Indeed, two loci that appear close in two dimensions may be further apart on the vertical axis. Confocal microscopy might aid in better assessing nuclear architecture in three dimensions.

Male infertility can be related to multiple factors. Regardless of the cause (e.g., high oxidative stress levels, radiation, hormonal alterations, genetic and chromosomal abnormalities), altered spermatozoa exhibit specific features: reduced nuclear condensation, initiation of an apoptosis process, abnormal responses to the hypo-osmotic swelling test, higher methylation levels, altered transcription, and abnormal nuclear architecture [36]. It was found that sperm telomere length increased with advancing paternal age and mitochondrial dysfunction, as well as nuclear DNA damage, providing new insights into male reproduction processes [38].

Here, we observed that high-magnification sperm morphology indirectly allows the detection of spermatozoa with an abnormal nuclear architecture. We, therefore, might suggest that high magnification allows the identification of high-potential spermatozoa, independently of the fertility etiology.

## Data Availability

Data are contained within the article.
